# Association between Head and Neck Cancer and Increased Risk of Ischemic Heart Disease: A Retrospective Cohort Study Using National Population Data

**DOI:** 10.3390/cancers16071352

**Published:** 2024-03-29

**Authors:** Chulho Kim, Hyunjae Yu, Dong-Kyu Kim

**Affiliations:** 1 Department of Neurology, Chuncheon Sacred Heart Hospital, Hallym University College of Medicine, Chuncheon 24253, Republic of Korea; gumdol52@hallym.or.kr; 2 Division of Big Data and Artificial Intelligence, Institute of New Frontier Research, Hallym University College of Medicine, Chuncheon 24253, Republic of Korea; 3 Department of Otorhinolaryngology-Head and Neck Surgery, Chuncheon Sacred Heart Hospital, Hallym University College of Medicine, Chuncheon 24253, Republic of Korea

**Keywords:** cancer, head and neck, ischemic heart disease, cardiovascular, cohort

## Abstract

**Simple Summary:**

This study elucidated an augmented incidence of ischemic heart disease (IHD) events in subjects diagnosed with head and neck cancer (HNC). The statistically significant escalation in the risk of IHD manifestation became apparent following a period of four years after the diagnosis of HNC, with a sustained high risk thereafter. Notably, an increased association with IHD events was observed among males with HNC, as well as in cases of malignancies localized to the oral cavity and sinonasal regions. Consequently, we advocate for the importance of using proactive strategies for early detection in the management of patients with HNC.

**Abstract:**

Although cancer and ischemic heart disease (IHD) frequently manifest in the same individual, the risk of IHD events in cancer, especially head and neck cancer (HNC), remains unclear. We aimed to examine the incidence and risk of IHD events in patients with HNC using a population-based cohort dataset in South Korea (2002–2013). Through rigorous propensity score matching, we compared data from 2816 individuals without HNC and 704 individuals with HNC. Key independent variables were matched between groups, and the Charlson Comorbidity Index was used to match comorbidities. The Kaplan–Meier method depicted the cumulative probability of IHD throughout the follow-up period for both the study and control groups. The overall IHD incidence was significantly higher (19.93) in patients with HNC than in those without HNC (14.81), signifying an augmented IHD risk in the HNC cohort. Subsequent temporal analysis revealed a significant surge in IHD risk commencing 4 years after HNC diagnosis and persisting throughout the follow-up period. Subgroup analysis revealed an increased IHD risk in men with HNC and patients with cancers affecting the oral and sinonasal regions. This retrospective cohort study provides valuable scientific insights into the nuanced relationship between HNC and IHD, underscoring the need for tailored monitoring protocols and specialized care for susceptible individuals.

## 1. Introduction

Globally, the incidence of cancer has been increasing in recent years [1,2,3] with varying mortality rates [4]. Despite the positive influence of improved treatment modalities, early detection strategies, and advancements in medical care [5,6], certain challenges and the prevalence of certain risk factors continue to affect cancer outcomes. Ischemic heart disease (IHD) is a cardiovascular disorder characterized by a reduced blood supply to the heart muscle due to the narrowing or blockage of the coronary arteries. It is a major cause of morbidity and mortality globally, and various risk factors, such as hypertension, diabetes, smoking, and high cholesterol levels, contribute to its development [7].

Several studies have demonstrated that cancer survivors have a higher incidence and risk of IHD than the general population [8,9,10]. Although IHD and cancer have distinct pathophysiological mechanisms and affect different organ systems, these often occur in the same patient owing to the common biological pathways and risk factors such as obesity, diabetes, and smoking [11,12], as well as inflammatory and hypercoagulable conditions often associated with many cancer types [13,14]. Moreover, certain cancer treatments have been identified as potential accelerators of atherosclerosis, contributing to the observed increase in IHD prevalence [15,16,17,18]. Nevertheless, identifying IHD at an early stage in cancer survivors can pose challenges, and effectively managing IHD in patients with cancer can frequently be complicated by factors associated with ongoing or prior cancer treatment, as well as underlying cancer itself.

Head and neck cancer (HNC) encompasses a range of malignancies affecting the oral cavity, pharynx, hypopharynx, larynx, sinonasal cavity, and salivary glands. Established risk factors for HNC include habitual tobacco use, alcohol consumption, prolonged exposure to environmental pollutants, and infection with human papillomavirus or Epstein–Barr virus. The neoplastic transformation of mucosal epithelial cells in these diverse anatomical sites underscores the heterogeneity of HNC. Collectively, it is the seventh most frequently diagnosed type of cancer worldwide [19]. It is more prevalent in men than in women and has a higher incidence in adults aged >50 years old [3]. Despite the improved survival rates of patients with HNC owing to modern medicine [20], a few studies have reported a concurrent increase in the prevalence of comorbidities and adverse effects associated with cancer therapies, particularly increased susceptibility to cardiovascular events in patients with HNC undergoing chemoradiation therapy, increased risk of stroke and myocardial infarction in patients with HNC [21], and an increased risk of cardiovascular events with older age and the presence of diabetes at the time of HNC diagnosis [22]. Nonetheless, to date, no study has systematically examined the occurrence and risk of IHD as a complication in individuals with HNC. Consequently, this study aimed to conduct an in-depth analysis of the incidence and plausible risk factors associated with IHD in patients with HNC using a dataset that is representative of the entire national population.

## 2. Materials and Methods

### 2.1. Ethical Statement

This study was approved by the Institutional Review Board (IRB) of Hallym Medical University, Chuncheon Sacred Heart Hospital (protocol code: 2021-08-006). As patient data were extracted and provided to the principal investigator as anonymized data, the requirement for obtaining written informed consent was waived. However, the data supporting the findings of this study are fully documented and accessible within the article. Adherence to ethical principles was maintained throughout this study in accordance with the guidelines outlined in the Declaration of Helsinki.

### 2.2. Nationwide Population-Based Cohort Dataset

This longitudinal study used a nationwide population-based sample cohort dataset derived from de-identified national health claims data obtained from the Korean National Health Insurance Service. This dataset encompasses a wide array of medical conditions, enabling the thorough exploration of the potential interconnections between these specific health phenomena. The uniqueness of South Koreans’ identification numbers assigned at birth ensures that there is no overlap or omission of medical claims data within the dataset. Additionally, the healthcare provider database supplements the cohort by providing comprehensive information on healthcare facilities, including types, personnel, and equipment. This national database encompasses a broad spectrum of medical utilization details such as dates of death, hospital visits, outpatient care, and medication history. The cohort dataset, encompassing approximately 2.2% of the Korean population, includes 1,025,340 adults and has demonstrated excellent reliability in a prior verification study [23,24].

### 2.3. Study Design: A Retrospective Cohort Approach

To explore the potential effect of HNC on IHD, we delineated two distinct cohorts: a target cohort comprising individuals with HNC and a comparative cohort comprising those without HNC. Using a retrospective cohort design, we conducted a comparative analysis of the two groups using data retrospectively collected from patient records. This longitudinal investigation commenced with observations from a specific historical point in time, scrutinizing the outcomes occurring between that juncture and the present.

**Figure 1:**
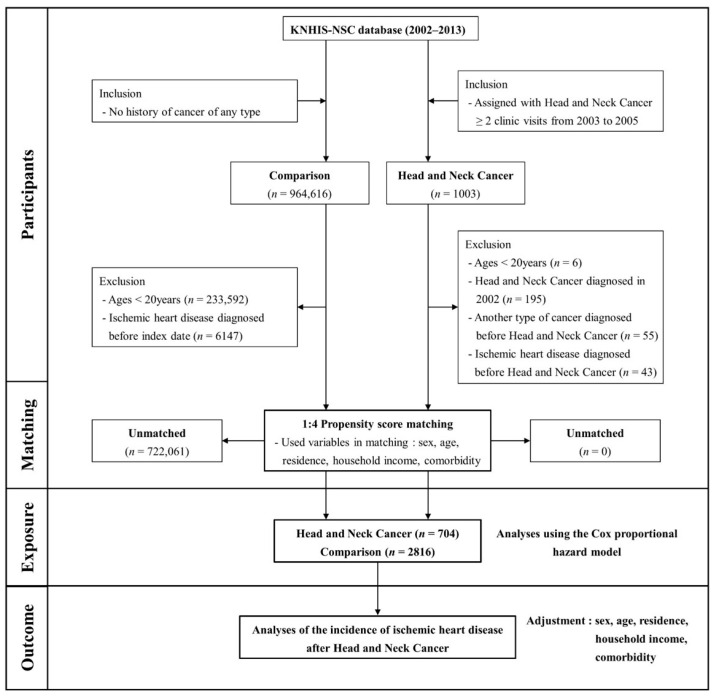
A brief illustration of the process of participant selection. KNHIS-NSC, Korean National Health Insurance Service-National Sample Cohort.

Figure 1 outlines the participant selection process. Patients with HNC were enrolled during the index period (2003–2005) and identified using diagnostic codes specific to various HNC types. For the comparative cohort, individuals without cancer were randomly selected from the remaining cohort within the database and matched to HNC patients using the propensity scoring methodology (four participants without cancer for each HNC patient). Key independent variables, including sex, age, residence, income level, and comorbidities, were meticulously matched between groups. The Charlson Comorbidity Index (CCI) was used to match comorbidities [25]. The CCI was first developed in 1987 by Mary Charlson and colleagues as a weighted index to predict the risk of death within 1 year of hospitalization for patients with specific comorbid conditions. Nineteen conditions were included in the index. A score of zero indicates that no comorbidities were found. The higher the score, the more likely the predicted outcome will result in mortality or higher resource use.

**Figure 2:**
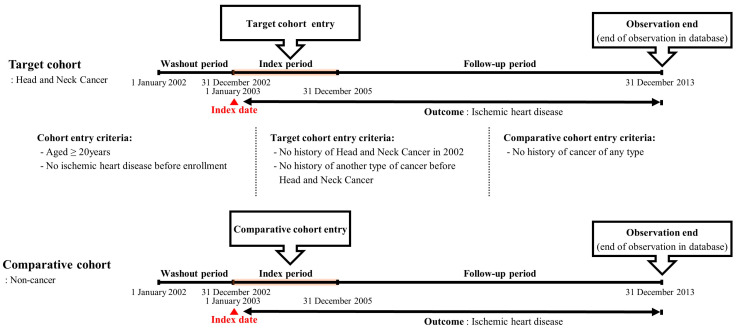
Description of the longitudinal retrospective cohort-based study design.

Figure 2 summarizes the retrospective cohort design. This study encompassed three distinct periods: washout, index, and observation. The washout period involved exclusion of data from the initial year (January–December 2002) of the cohort dataset to mitigate the presence of IHD events before the HNC diagnosis. Throughout the observation period, follow-up was concluded based on the occurrence of the primary outcome (IHD; I20–I25) or death of the participant. If patients in the database experienced no events during the final follow-up period, their data were censored at that time point.

### 2.4. Statistical Analysis

To determine the sample size of a retrospective cohort study, a minimum of 10 respective event cases in each cohort group should be included to ensure that the outcomes are statistically reliable and clinically relevant [26,27]. This guideline serves as a benchmark for maintaining the integrity and applicability of a study’s results. In scenarios where the optimal threshold cannot be met, it is considered permissible to lower the standard to a minimum of seven or even five occurrences per predictor variable. The primary focus of this study was to assess the incidence and hazard ratio (HR) of IHD in patients with HNC compared to those in patients without cancer. The overall IHD incidence was calculated by dividing the number of patients diagnosed with IHD by 1000 person-years. The period considered for this calculation spanned from the date of patient enrollment to the individual endpoint for each patient. To evaluate whether HNC could increase the HR for incident-specific disease events, we employed Cox’s proportional hazards regression. Outcomes were expressed as HR and 95% confidence intervals (CI). Both crude and adjusted HRs, accompanied by their respective 95% Cis, were reported for comprehensive analysis. Statistical analyses were conducted using the R software (version 4.0.0) from the R Foundation for Statistical Computing, Vienna, Austria. We used R packages such as matchit for propensity scoring matching, epiR for incidence analysis, survival analysis for Cox’s regression, and ggplot2 for figure drawing. P significance was set at *p* < 0.05.

## 3. Results

### 3.1. Comparing the Comparative and Target Cohorts

A 4:1 propensity scoring method used for matching the comparative and target cohorts revealed a strikingly similar distribution across all covariates in both cohorts and no significant differences in any independent variables between the two cohorts (Table 1).

**Figure 3:**
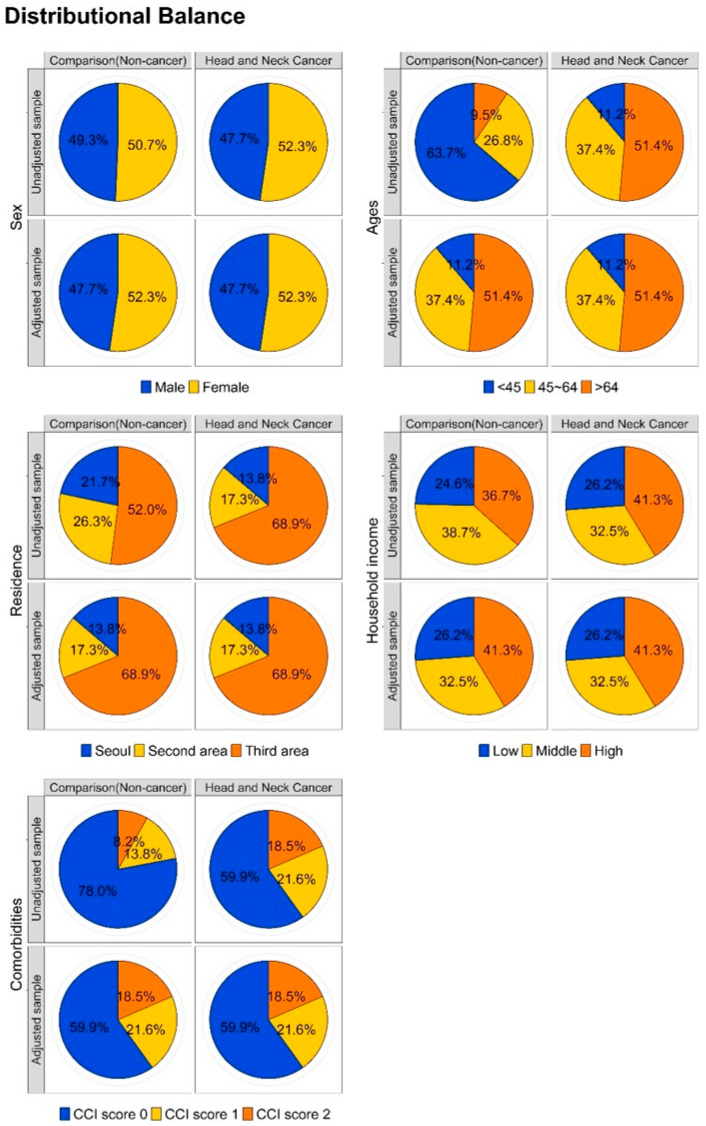
A balance plot used to visually assess the efficacy of the cohort matching process for five independent variables.

In addition, a balance plot test conducted to visually assess the efficacy of the matching process (Figure 3) demonstrated a harmonized distribution after matching, reinforcing the appropriateness and robustness of cohort matching between the comparative and target cohorts.

### 3.2. Effect of Head and Neck Cancer on Subsequent Ischemic Heart Disease Development

A comprehensive analysis of 23,563.2 person-years in the comparative cohort and 4817.7 person-years in the target cohort to assess the event rates and HRs for IHD during the follow-up period (Table 2) revealed an increased IHD incidence in patients diagnosed with HNC (19.93 vs. 14.81 of the control group). Additionally, the Cox regression analysis revealed a statistically significant increase in the overall adjusted risk for subsequent IHD development in patients, reaching 1.33 (95% CI = 1.06–1.67) during the follow-up period (Table 3). Interestingly, we found that the results obtained from the four models depending on the degree of adjusted variables were all similar.

Moreover, an in-depth examination of the risk during the follow-up period indicated that although the probability of IHD did not exhibit a significant increase during the initial 3 years after HNC diagnosis, a noteworthy increase in risk emerged from the fourth year after diagnosis (Table 4). Subsequently, the consistently significant adjusted risk of subsequent development of IHD persisted throughout the follow-up period.

### 3.3. Risk of Ischemic Heart Disease Events in Various Subgroups

**Figure 4:**
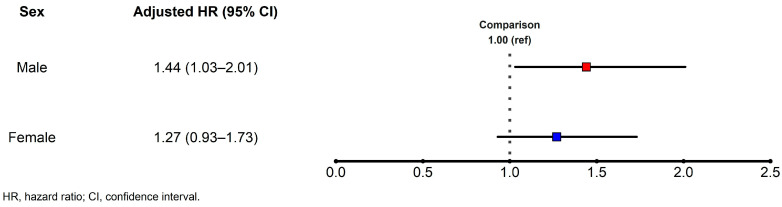
The hazard ratio of ischemic heart disease by sex in a comparison between patients with and without HNC. HNC, head and neck cancer (we used Model 4, which was adjusted for demographic factors and the Charlson comorbidity index).

When conducting a subgroup analysis based on sex, we noted an increased risk of IHD in male patients with HNC, whereas the adjusted HR of IHD in female patients with HNC was statistically insignificant (Figure 4). As a result of adding interaction to Model 4, there was no interaction effect (*p* = 0.596).

Additionally, to evaluate the risk of IHD according to the HNC subtype, we categorized the HNC group into subgroups, such as oral cavity, salivary gland, oropharynx, nasopharynx, hypopharynx, sinonasal tract, and larynx. Results from both the uni- and multivariable Cox regression models revealed that the oral cavity and sinonasal tract groups exhibited a significantly increased risk of IHD events at 1.40 (1.09–1.80) and 3.53 (1.12–11.13), respectively. In contrast, no statistically significant HR was observed for the other HNC subtypes (Table 5).

## 4. Discussion

Considering that cardiovascular disease ranks as the second largest cause of morbidity and mortality in cancer patients [27], numerous clinicians and researchers have examined the correlation between cancer and increased susceptibility to cardiovascular disease. This study offers insights into the correlation between the risk of IHD and HNC diagnosis. Using a robust nationwide population-based cohort dataset, our investigation revealed a distinct increase in incident IHD occurrence within the HNC patient cohort compared to that in patients without HNC. Stringent adjustments for covariates, including sex, age, residence, income level, and comorbidities (Model 1 to 4), consistently demonstrated a statistically significant increase in the adjusted HR for IHD within the HNC population during the follow-up period and approximately 4 years after HNC diagnosis. Moreover, IHD incidence was higher in men and those with cancers of the oral cavity and sinonasal tract. However, it is essential to acknowledge a potential limitation: the accuracy of disease diagnoses in the claims data may not perfectly reflect the true health status of the patients. To address this concern and enhance diagnostic precision, there is a recognized need for a carefully defined operational definition of diagnoses.

This study represents a pioneering effort to systematically examine the risk landscape associated with IHD incidence in individuals diagnosed with HNC. Several known conventional risk factors, including race, advanced age, diabetes mellitus, hypertension, and cancer-related factors such as human papillomavirus status and radiation therapy, are correlated with the risk of cardiovascular disease in patients with HNC [22,28,29,30]. Additionally, to enhance the power of analysis, we adjusted two cohort groups according to the selected independent variables. However, this cohort dataset provided grouped patient age distributions due to de-identification issues. Therefore, it had the limitation of not being able to determine the actual age of each patient. Moreover, comorbidity status was controlled by the CCI score, not the specific disease. Thus, we could not completely remove the selection bias issue. Consequently, our findings suggested that the increased risk of IHD may be attributable not only to the variables associated with personal factors but also to the inherent characteristics of HNC. Moreover, our observations revealed that during the follow-up period, a significant increase in risk did not become apparent until the fourth year after diagnosis. Subsequently, a consistently significant adjusted HR for the subsequent development of IHD persisted throughout the follow-up period. That the risk of IHD increases significantly after the cancer has been present for some time suggests that cancer itself may be a risk factor for IHD.

HNC exhibits a discernible sex-based predilection, with a higher incidence in men. Historically, men have exhibited a higher prevalence of tobacco and alcohol consumption, which are the established etiological agents of HNC. The synergistic effect of concomitant smoking and heavy alcohol use amplifies susceptibility to these cancers, and the historical proclivity of men toward these behaviors accentuates the sex-based prevalence differential [31,32]. Furthermore, occupations associated with increased exposure to industrial compounds or environmental carcinogens, which are more prevalent in men, may augment the risk profile for this malignancy [33,34]. Although the precise mechanisms remain unclear, emerging evidence suggests that hormones, particularly estrogen in women, may exert a protective effect against specific HNC subtypes [35,36]. Consistent with these findings, a sex-based subgroup analysis in this study revealed that male patients with HNC exhibited a relatively higher hazard than the control subjects, whereas the HR of incident IHD events was not statistically significant in female patients with HNC. Furthermore, when evaluating the risk of IHD based on the HNC subtype, we observed that the oral cavity and sinonasal tract groups exhibited a significantly increased risk of IHD events, consistent with previous reports [37,38,39]. However, the number of included patients and the number of IHD events in each HNC subtype is minimal, and except for the oral cavity cancer group, there is a limitation in terms of identifying significant clinical implications about the data via statistical analysis.

Our study has several methodological strengths. Firstly, nationwide population-based data were used to investigate IHD incidence in patients with HNC. Thus, the propensity score matching method enabled the effective control of critical confounding variables, facilitating a meticulous comparison of IHD incidence between the HNC cohort and a concisely matched control group. Our findings provide implications for clinical practice regarding the potential associations between HNC and IHD. Secondly, this study had a robust design, encompassing a substantial patient cohort and an extensive 11-year observation period. This prolonged timeframe allowed for a comprehensive examination of the temporal relationship between HNC diagnosis and the subsequent development of IHD. Thirdly, to attenuate surveillance bias in assessing IHD risk in patients with HNC, meticulous efforts were undertaken to select sociodemographically matched controls from the cohort database. This approach, ensuring that the control group shares similar sociodemographic characteristics with patients with HNC, aimed to minimize potential bias related to surveillance practices, thereby enhancing the reliability of the findings and providing a more accurate evaluation of the association between HNC and the risk of IHD. Fourthly, given the possibility of underdiagnosing IHD in patients with cancer due to cancer management, a wash-out period of one year before the index period was implemented in our research design. This measure aimed to minimize the length of time bias, contributing to the robustness of our findings. Finally, we systematically assessed variations in the risk dynamics of IHD development across increasing observation periods. This study aimed to determine whether the observed risk of IHD in individuals with HNC was a coincidental occurrence confined to specific temporal intervals or manifested as a sustained phenomenon following a discernible pattern. Our findings revealed a sustained increase in the long-term risk of developing an IHD after an HNC diagnosis. This observation strongly suggests a substantive association between HNC and the occurrence of IHD, indicating a nonrandom correlation rather than a chance event.

However, this study had a few limitations. Primary disease identification was based on diagnostic codes derived from the International Classification of Diseases, Tenth Revision, Clinical Modification, rather than an exhaustive examination of individual medical records. This methodology precludes access to crucial details including comprehensive medical histories and pathological reports, thereby impeding a nuanced understanding of the intricacies associated with the conditions under investigation. Consequently, the analysis was deprived of the requisite granularity necessary to account for pivotal factors, such as cancer staging and severity of IHD, resulting in a constrained depth of comprehension. Next, we drew a cohort sample database featuring a delimited set of identifiable variables. This constraint hindered the inclusion of certain potentially confounding risk factors influencing the occurrence of IHD, including family medical history, smoking history, and the quantification of alcohol consumption. The absence of comprehensive data about these variables introduced confounding factors in the analysis. Additionally, owing to the unavailability of information regarding treatment modalities for HNC, this study was unable to assess the impacts of therapeutic interventions such as chemotherapy and radiotherapy on the risk of IHD. Insights into the influence of these treatments may offer valuable insights into the intricate relationship between cancer and cardiovascular conditions. Finally, the retrospective cohort design of the study restricted the direct exploration and analysis of the pathological mechanisms underlying the relationship between HNC and IHD. Subsequent clinical investigations that incorporate a more extensive array of factors and adopt a prospective design are imperative to unravel the intricate pathophysiological mechanisms linking these complex conditions.

## 5. Conclusions

This study revealed a higher incidence of IHD events in individuals diagnosed with HNC. Notably, a statistically significant increase in the risk of IHD development manifested after a 4-year post-HNC diagnosis period, maintaining a high risk thereafter. Of particular interest was the discerned elevated association with IHD events among men with HNC and those with malignancies localized to the oral cavity and sinonasal regions. These findings provide novel and substantive insights into the complex relationship between HNC and IHD. Therefore, we suggest encouraging heightened clinical awareness among healthcare practitioners, emphasizing the need for proactive measures for early detection in the management of patients with HNC.

## Figures and Tables

**Table 1:** Characteristics of this study subjects.

Variables	Comparative Cohort (n = 2816)	Target Cohort (n = 704)	*p*-Value
Sex			1.000
Male	1344 (47.7%)	336 (47.7%)	
Female	1472 (52.3%)	368 (52.3%)	
Ages (years)			1.000
<45	316 (11.2%)	79 (11.2%)	
45–64	1052 (37.4%)	263 (37.4%)	
≥64	1448 (51.4%)	362 (51.4%)	
Residence			1.000
Seoul	388 (13.8%)	97 (13.8%)	
Second area	488 (17.3%)	122 (17.3%)	
Third area	1940 (68.9%)	485 (68.9%)	
Household income			1.000
Low (0–30%)	736 (26.1%)	184 (26.1%)	
Middle (30–70%)	916 (32.5%)	229 (32.5%)	
High (70–100%)	1164 (41.3%)	291 (41.3%)	
CCI			1.000
0	1688 (59.9%)	422 (59.9%)	
1	608 (21.6%)	152 (21.6%)	
≥2	520 (18.5%)	130 (18.5%)	

Seoul, largest metropolitan area; second area, other metropolitan cities; third area, other areas; CCI, the Charlson Comorbidity Index. We calculated the *p*-value using Pearson’s Chi-squared test.

**Table 2:** The overall incidence of ischemic heart disease events in patients with head and neck cancer during the follow-up period.

Variables	N	Case	Median Follow-Up Period	Person Year	Incidence
Comparative cohort	2816	349	8.997 years	23,563.2	14.81
Target cohort	704	96	8.329 years	4817.7	19.93

**Table 3:** The hazard ratio of ischemic heart disease events in patients with head and neck cancer during the follow-up period.

HR (95%CI)	Model 1	Model 2	Model 3	Model 4
Comparative cohort	1.00 (ref)	1.00 (ref)	1.00 (ref)	1.00 (ref)
Target cohort	1.33 (1.06–1.67)	1.34 (1.07–1.68)	1.34 (1.07–1.68)	1.33 (1.06–1.67)

HR, hazard ratio; CI, confidence interval. Model 1: crude HR; Model 2: adjusted for age and sex; Model 3: adjusted for demographic factors (age, sex, residence, and income level); Model 4: adjusted for demographic factors and the Charlson comorbidity index.

**Table 4:** Risk of ischemic heart disease events by time elapsed since the diagnosis of head and neck cancer.

Time (Year)	Ischemic Heart Disease	Ischemic Heart Disease
	Model 1 (95% CI)	Model 4 (95% CI)
1	1.07 (0.61–1.90)	1.08 (0.61–1.90)
2	1.20 (0.79–1.83)	1.21 (0.79–1.83)
3	1.38 (0.97–1.96)	1.38 (0.97–1.97)
4	1.48 (1.10–2.01)	1.49 (1.10–2.02)
5	1.35 (1.01–1.81)	1.36 (1.02–1.81)
6	1.31 (1.00–1.72)	1.32 (1.00–1.73)
7	1.43 (1.11–1.84)	1.44 (1.12–1.85)
8	1.39 (1.09–1.77)	1.39 (1.09–1.77)
9	1.40 (1.11–1.77)	1.40 (1.12–1.77)
10	1.36 (1.08–1.70)	1.36 (1.08–1.71)
11	1.33 (1.06–1.67)	1.33 (1.06–1.67)

HR, hazard ratio; CI, confidence interval. Model 1: crude HR; Model 4: adjusted for demographic factors and the Charlson comorbidity index.

**Table 5:** Incidence rate and hazard ratio for developing ischemic heart disease according to the subtype of head and neck cancer.

Variables	N	Case	Person Year	Incidence Rate	Model 1 (95% CI)	Model 2 (95% CI)
Cancer type						
Comparison	2816	349	23,563.2	14.81	1.00 (ref)	1.00 (ref)
Oral cavity	495	76	3489.4	21.78	1.46 (1.14–1.87)	1.40 (1.09–1.80)
Salivary gland	23	3	144.8	20.71	1.37 (0.44–4.28)	2.37 (0.75–7.48)
Oropharynx	24	3	169.6	17.69	1.18 (0.38–3.69)	1.29 (0.41–4.04)
Nasopharynx	39	2	232.5	8.60	0.57 (0.14–2.29)	0.71 (0.18–2.86)
Hypopharynx	13	1	87.8	11.40	0.76 (0.11–5.43)	0.66 (0.09–4.69)
Sinonasal tract	16	3	91.8	32.69	2.16 (0.69–6.74)	3.53 (1.12–11.13)
Larynx	94	8	601.9	13.29	0.89 (0.44–1.79)	0.89 (0.44–1.80)

HR, hazard ratio; CI, confidence interval. Model 1: crude HR, Model 2: adjusted for age and sex.

## Data Availability

The datasets generated and/or analyzed in the current study are not publicly available owing to the policy of the Korea National Health Insurance Service, but they are available from the corresponding author upon reasonable request.
